# EBV-miR-BART8-3p induces epithelial-mesenchymal transition and promotes metastasis of nasopharyngeal carcinoma cells through activating NF-κB and Erk1/2 pathways

**DOI:** 10.1186/s13046-018-0953-6

**Published:** 2018-11-26

**Authors:** Cheng Lin, Jingfeng Zong, Wansong Lin, Minghui Wang, Yuanji Xu, Rui Zhou, Shaojun Lin, Qiaojuan Guo, Honglin Chen, Yunbin Ye, Bin Zhang, Jianji Pan

**Affiliations:** 1 Fujian Medical University, Fuzhou, 350108 Fujian Province China; 2 Department of Radiation Oncology, Fujian Cancer Hospital & Fujian Medical University Cancer Hospital, Fuzhou, 350011 Fujian Province China; 3 Laboratory of Immuno-Oncology, Fujian Cancer Hospital & Fujian Medical University Cancer Hospital, Fuzhou, 350011 Fujian Province China; 4 Department of Genetics and Genomic Sciences, Icahn Institute of Genomics and Multiscale Biology, Mount Sinai Center for Transformative Disease Modeling, Icahn School of Medicine at Mount Sinai, 1470 Madison Avenue, New York, NY 10029 USA; 5 State Key Laboratory for Emerging Infectious Diseases, Department of Microbiology and the Collaborative Innovation Center for Diagnosis and Treatment of Infectious Diseases, The University of Hong Kong, Hong Kong, SAR China; 6 Fujian Provincial Key Laboratory of Translational Cancer Medicine, Fuzhou, 350014 Fujian China

**Keywords:** Nasopharyngeal carcinoma, Epithelial-mesenchymal transition, Metastasis, Epstein-Barr virus, EBV-miR-BART8-3p, NF-κB signaling, Erk1/2 signaling

## Abstract

**Background:**

Epstein-Barr virus (EBV) is ubiquitously associated with nasopharyngeal carcinoma (NPC). EBV encodes two groups of microRNAs (miRNAs) which are divided into *Bam*HI fragment H rightward open reading frame 1 (BHRF1) and BamHI-A rightward transcripts (BART) microRNAs. EBV miR-BART has been found to be involved in the development and progression of NPC. However, so far the role of EBV-miR-BART8-3p in NPC progression remains unknown. This study aimed to investigate the role of EBV-miR-BART8-3p in NPC and explore the underlying mechanisms.

**Methods:**

miRNA expression was profiled in NPC and normal nasopharyngeal mucosal specimens using miRNA sequencing. EBV-miR-BART8-3p and RNF38 expression was quantified with qPCR assay. The migration, invasion and metastasis of NPC cells were evaluated using CCK-8, colony-forming, wound-healing, and migration and invasion assays. The expression levels of epithelial-mesenchymal transition (EMT)-related markers,metastasis-related markers and NF-κB and Erk1/2 signaling proteins were determined using Western blotting. Tumorigenic assay was performed to evaluate the pulmonary metastatic ability of NPC cells in vivo.

**Results:**

EBV BART miRNAs were highly over-expressed and co-expressed in NPC and might be associated with deactivated immune response in NPC according to the sequencing analysis. EBV-miR-BART8-3p expression was significantly higher in human NPC specimens than in normal nasopharyngeal mucosal specimens. EBV-miR-BART8-3p was found to promote NPC migration, invasion and metastasis, drove an EMT process and upregulated expression of metastasis-related proteins expression in NPC cells. Our data showed EBV-miR-BART8-3p directly targeted RNF38 in NPC cells.

**Conclusion:**

The present study demonstrates that EBV-miR-BART8-3p plays a significant role in inducing EMT and promoting metastasis through directly targeting RNF38 in NPC cells via the activation of NF-κB and Erk1/2 signaling pathways. Our findings suggest that EBV-miR-BART8-3p is a potential therapeutic target for NPC.

**Electronic supplementary material:**

The online version of this article (10.1186/s13046-018-0953-6) contains supplementary material, which is available to authorized users.

## Background

Nasopharyngeal carcinoma (NPC) is the most common primary malignancy in the nasopharynx [1, 2]. Although NPC is rare worldwide [3], this malignancy remains highly prevalent in endemic regions, notably in southern China [4]. Currently, radiotherapy remains the treatment of choice for NPC, and concomitant chemoradiotherapy has been proved to increase the survival [5, 6]. Although 80 to 90% 5-year local control rates have been achieved for NPC, there are still 15 to 30% of patients developing distant metastasis [7]. Elucidation of the mechanisms underlying NPC metastasis in NPC is therefore of great significance to improve the prognosis and treatment outcomes.

Epstein-Barr virus (EBV) has been accepted as an etiological factor for NPC. Almost 100% of non-keratinizing NPC, are associated with EBV infections [1], which accounts for 95% of all NPCs in endemic regions [2]. A set of EBV latent genes have been identified to play an important role in the development of NPC, including three latent membrane proteins (LMP1, LMP2A and LMP2B), EBV nuclear antigen 1 (EBNA1) and EBV-encoded RNAs (EBERs) [3]. However, only three latent proteins (EBNA1, LMP1 and LMP2A) are expressed in type II latency of EBV infection, a predominant form of latency observed in NPC [4]. Whereas EBV-encoded microRNAs (EBV-miRNAs) are detected in most of clinical NPC specimens and cells during all the forms of latency [5–8].

EBV is the first identified human tumor-causing virus that encodes miRNAs. EBV-miRNAs consist of more than 10% of total miRNAs in NPC specimens and EBV-positive NPC C666–1 cells, and increasing evidence shows that EBV-miRNAs contribute to cancer survival [9–13], metastasis [14–18], immune evasion [19, 20] and latency [21–24]. Currently, EBV-miRNAs are divided into two clusters based on their locations, including *Bam*HI fragment H rightward open reading frame 1 (BHRF1) and *Bam*HI-A rightward transcripts (BART) miRNAs. EBV BART miRNAs contain 22 miRNA precursors (termed EBV-miR-BART1 to 22) that produce totally 44 mature miRNAs [6]. Previous studies showed that EBV BART miRNAs were highly overexpressed in clinical specimens and plasma samples of NPC patients and were involved in the development and progression of NPC [5, 25]. However, so far the role of EBV-miR-BART8-3p in NPC progression remains unknown. This study aimed to investigate the role of EBV-miR-BART8-3p in NPC, and explore the underlying mechanisms.

## Materials and methods

### Ethical approval

This study was approved by the Ethical Review Committee of Fujian Cancer Hospital (approval no. FJZLYY2016–00143). Written informed consent was obtained from all participants following a detailed description of the purpose of the study. All experiments described in this study were conducted in accordance with international and national laws, regulations and guidelines.

### Clinical specimens

Six EBV-positive NPC biopsy specimens and 4 normal nasopharyngeal mucosal specimens were sampled for miRNA sequencing, and another 19 NPC specimens and 10 normal nasopharyngeal specimens were used to quantify EBV-BART8-3p and RNF38expression. All NPC was diagnosed by pathological examinations, and all specimens were stored in liquid nitrogen for the subsequent experiments.

### Animals

Five-week-old female BALB/c nude mice were purchased from the Medical Experimental Animal Center of Guangdong Province (Guangzhou, China). All mice were housed in a clean facility in the Laboratory Animal Center of Fujian Medical University (Fuzhou, China) and given free access to clean water and food.

### Cell lines and culture

Two EBV-negative NPC cell lines CNE-1 and SUNE-1 and human embryonic kidney (HEK) 293 T cells were purchased from China Center for Type Culture Collection (Wuhan, China), and one EBV-positive C666–1cell line was kindly presented by Professor Hong lin Chen from the University of Hong Kong. All NPC cells were cultured in RPMI-1640 medium (HyClone; Logan, UT, USA) supplemented with 10% fetal bovine serum (FBS; Gibco, Grand Island, NY, USA) at 37 °C containing 5% CO_2_.HEK293T cells were maintained in Dulbecco’s modified Eagle’s medium (DMEM; HyClone, Logan, UT, USA) supplemented with 10% FBS at 37 °C containing 5% CO_2_.

### RNA sequencing and data analysis

Paired-end (2 × 150 bp) RNA-seq assays were performed from ribosomal RNA depleted total RNA on Illumina HiSeq 2500 system (Illumina; San Diego, CA, USA) according to the standard manufacturer’s protocol. The raw sequence reads were aligned to human genome GRCh38 with the star aligner (v2.5.0b) [26]. Then the gene level expression was quantified by feature Counts (v1.4.4) [27] based on GENCODE gene model version p2 release22. Genes with at least 1 count per million (CPM) in at least 1 sample were kept other removed from further analysis. The gene level read counts data was normalized using the trimmed mean of M-values normalization (TMM) method [28] to adjust for sequencing library size difference. Differential gene expression between cancer and control groups was predicted by a linear model analysis using Bioconductor package limma [29]. To adjust for multiple tests, the false discovery rate (FDR) of the differential expression test was estimated using the Benjamini-Hochberg method [30]. Genes with FDR adjusted *p* value ≤0.05 and fold change (FC) ≥ 1.2 were considered significant. Heatmap and cluster dendrogram of the significant genes were plotted using R programming language (https://www.r-project.org/). Gene ontology (GO) and pathways enriched in the differentially expressed genes were identified by Fisher’s exact test (FET) based on the gene set annotation collections from MSigDB [31].

### miRNA sequencing and data analysis

Total RNA was extracted from NPC specimens and normal nasopharyngeal mucosal specimens using TRIzol reagent (Invitrogen; Carlsbad, CA, USA). The RNA concentration was measured with a NanoDrop2000 Spectrophotometer (NanoDrop Technologies; Wilmington, DE, USA), and the integrity of purified RNA was determined using Agilent 2100 Bioanalyzer (Agilent Technologies; Palo Alto, CA USA). miRNA quantification was evaluated using Hot Start PCR. A small RNA library was built using the Next Multiplex Small RNA Library Prep Set for Illumina (NEB; Ipswich, MA, USA) according to the manufacturer’s protocol. Polyacrylamide gel electrophoresis was performed to purify small RNA and to enrich for molecules ranging from 18 to 30 nt. Then, cDNA was synthesized, digested and amplified to set cDNA libraries, followed by purification on a polyacrylamide gel and quantification. Finally, the cDNA libraries were sequenced using standard protocols on an Illumina Hiseq 4000 System (Illumina; San Diego, CA, USA). miRNA annotation was performed in the miRBase database (http://www.mirbase.org). Sequencing data were aligned to the reference human genome (UCSC hg19) and EBV genome (GCF_000872045.1).

Reads mapped to known miRNAs were identified by searching miRBase database (v21). Novel miRNAs were predicted by miRDeep [32]. Known and novel miRNA expression levels were measured by the number of mapped reads. Similar to the gene level differential expression analysis, differentially expressed miRNAs between cancer and control groups were predicted by limma following library depth normalization by the TMM method.

### microRNA co-expression network analysis

The weighted network analysis begins with a matrix of the Pearson correlations between all miRNA pairs, then converts the correlation matrix into an adjacency matrix using a power function f(x) = x^β. The parameter β of the power function is determined in such a way that the resulting adjacency matrix (i.e., the weighted co-expression network), is approximately scale-free. To measure how well a network satisfies a scale-free topology, we use the fitting index [33] (i.e., the model fitting index *R*^2^ of the linear model that regresses log(*p*(k)) on log(k) where k is connectivity and *p*(k) is the frequency distribution of connectivity). The fitting index of a perfect scale-free network is 1. For this dataset, we select the smallest β which leads to an approximately scale free network. The distribution *p*(k) of the resulting network approximates a power law: *p*(*k*) ∼ *k*^−*γ*^.

To explore the modular structures of the co-expression network, the adjacency matrix is further transformed into a topological overlap matrix [33]. As the topological overlap between two genes reflects not only their direct interaction, but also their indirect interactions through all the other genes in the network, previous studies have shown that topological overlap leads to more cohesive and biologically meaningful modules [33, 34]. To identify modules of highly co-regulated microRNAs, we use average linkage hierarchical clustering to group genes based on the topological overlap of their connectivity, followed by a dynamic cut-tree algorithm to dynamically cut clustering dendrogram branches into miRNA modules [35]. To distinguish between modules, each module was assigned a unique color identifier, with the remaining, poorly connected miRNAs colored grey.

### qPCR assay

The EBV-miR-BART8-3p and RNF38 expression were quantified using qPCR assay. Briefly, total RNA was extracted from NPC biopsy specimens and normal nasopharyngeal mucosal specimens using TRIzol reagent (Invitrogen; Carlsbad, CA, USA), and transcribed into cDNA using miScript II RT Kit (Qiagen; Valencia, CA, USA) following the manufacturer’s instructions. qPCR assay was performed using the miScript SYBR Green PCR Kit (Qiagen). *U6* snRNA and *GAPDH* were served as internal controls for quantifying miRNA and mRNA expression, respectively. The relative quantity of miRNA and mRNA expression was calculated using the 2^−ΔΔCt^method. All determinations were repeated in triplicate.

### Lentiviral transfection

Lentiviral particles (GV369 and Ubi-MCS-SV40-EGFP-IRES-puromycin) containing EBV-miR-BART8-3p precursors and lentiviral particles (GV280 and hU6-MCS-Ubiquitin-EGFP-IRES-puromycin) containing reverse complement of EBV-miR-BART8-3p and their control vectors were constructed by Shanghai Genechem Co., Ltd. (Shanghai, China).CNE-1 and SUNE-1 cells were transfected with a recombinant lentiviral vector GV369 to upregulate EBV-miR-BART8-3p expression (CNE-1-BART8-3p and SUNE-1-BART8-3p cells), and C666–1 cells were transfected with a lentiviral vector GV280 to downregulate EBV-miR-BART8-3p expression (C666–1-BART8-3p cells). The transfection efficiency was checked using qPCR assay.

For the rescue assay, CNE-1-BART8-3p cells and SUNE-1-BART8-3p cells were transfected with the RNF38 lentiviral vector GV358 (Shanghai Genechem Co., Ltd.; Shanghai, China) or a normal control (Shanghai Genechem Co., Ltd.; Shanghai, China).

### Cell proliferation and colony-forming assays

For cell proliferation assays, cells were seeded onto 96-well plates (Corning, Inc.; Corning, NY, USA) at a density of 1500cells per well and were incubated at 37 °C containing 5% CO_2_ for 1, 2 and 3 days. Subsequently, 10 μl of Cell Counting Kit-8 solution (Dojindo; Dojindo Molecular Technologies, Inc., Tokyo, Japan) was added to each well and incubated for 2 h. Then, the absorbance value (OD) was measured at 450 nm.

For colony-forming assays, cells were seeded onto 6-well plates (Corning, Inc.; Corning, NY, USA) at a density of 1000 cells per well and each group had three replicate wells. Following 2-week incubation, cell colonies were washed twice in PBS, fixed with 100% methanol and stained with crystal violet staining solution (Beyotime Institute of Biotechnology; Shanghai, China) for 15 min for counting.

### Wound healing assay

Cells were seed onto 6-well plates (Corning, Inc.; Corning, NY, USA), and artificial wounds were created using a sterile 200 μl plastic tip following serum starvation for 24 h. Then, cells were washed with serum-free medium to remove debris and floating cells. Images of the wounds were captured at 0, 24 and 48 h under an inverted microscope.

### Cell migration and invasion assays

For cell migration and invasion assays, cells in serum-free medium were transferred to the upper chamber of 8.0 μm pore size (Corning, Inc.; Corning, NY, USA) without or with Matrigel (BD Biosciences, Franklin Lakes, NJ, USA), and 20% FBS was added to the bottom chamber. SUNE-1 and CNE-1 cells were incubated in the upper chamber and removed 24 (cell migration assays) or 48 h after incubation (cell invasion assays), respectively, while C666–1 cells were incubated for 48 h in migration and invasion assays. Then, cells on the lower surface of the membrane were fixed and stained. Finally, images were captured and cell numbers were counted using microscopy (100 ×).

### Western blotting analysis

Western blotting analyses were performed by using standard protocols. Primary antibodies, including rabbit anti-RNF38 polyclonal antibody (1:250; Abcam, Cambridge, MA, USA), rabbit anti-Vimentin monoclonal antibody (1:1000; Abcam), rabbit anti-E-cadherin monoclonal antibody (1:1000; Abcam), rabbit anti-Snail monoclonal antibody (1:1000; Cell Signaling Technology, Inc.), rabbit anti-N-cadherin monoclonal antibody (1:5000; Abcam), rabbit anti-MMP2 polyclonal antibody (1:200; Santa Cruz Biotechnology, Inc.; Santa Cruz, CA, USA), rabbit anti-MMP9 monoclonal antibody (1:1000; Abcam), mouse anti-IKKα monoclonal antibody (1:200; Santa Cruz Biotechnology, Inc.), rabbit anti-p-IKKα/β monoclonal antibody (1:1000; Cell Signaling Technology, Inc.; Danvers, MA, USA), rabbit anti-IKKβ monoclonal antibody (1:1000; Cell Signaling Technology, Inc.), rabbit anti-IκBɑ monoclonal antibody (1:1000; Cell Signaling Technology, Inc.), rabbit anti-p-IκBɑ monoclonal antibody (1:1000; Cell Signaling Technology, Inc.), mouse anti-IκBβ monoclonal antibody (1:200; Santa Cruz Biotechnology, Inc.), rabbit p-IκBβ monoclonal antibody (1:1000; Cell Signaling Technology, Inc.), mouse anti-NF-κB monoclonal antibody (1:200; Santa Cruz Biotechnology, Inc.), rabbit anti-p-NF-κB monoclonal antibody (1:1000; Cell Signaling Technology, Inc.), rabbit anti-Erk1/2 monoclonal antibody (1:1000; Cell Signaling Technology, Inc.), rabbit anti-p-Erk1/2 monoclonal antibody (1:1000; Cell Signaling Technology, Inc.), rabbit anti-p-Mek1/2 monoclonal antibody (1:1000; Cell Signaling Technology, Inc.), rabbit anti-p-c-Raf monoclonal antibody (Ser259) (1:1000; Cell Signaling Technology, Inc.), rabbit anti-TAK1 monoclonal antibody (1:1000; Cell Signaling Technology, Inc.), and rabbit anti-p-TAK1 monoclonal antibody (1:1000; Cell Signaling Technology, Inc.) were used. Membranes were incubated with the primary antibodies at 4 °C overnight, followed by incubation with the horseradish peroxidase (HRP)-conjugated secondary anti-rabbit or anti-mouse IgG antibody (1:3000; Santa Cruz Biotechnology, Inc.) for 1 h at room temperature, while rabbit anti-GAPDH polyclonal antibody (1:10000; Abcam) served as a loading control.

### Bioinformatics analysis and dual-luciferase reporter assay

The potential target genes of EBV-miR-BART8-3p were predicted using two publicly available algorithms RepTar (http://reptar.ekmd.huji.ac.il/) and DIANA TOOLS (http://diana.imis.athena-innovation.gr/DianaTools/index.php?r=tarbase/index). Then, the predicted candidate targets were confirmed by the software RNAhybrid (https://bibiserv.cebitec.uni-bielefeld.de/) with low minimum free energy (MFE) (≤ − 20.0). To validate whether RNF38 was a direct target of EBV-miR-BART8-3p, wide-type or mutated luciferase reporter vectors of RNF38 were transfected into HEK293T cells with a miR-BART8-3p mimic or normal control. The Firefly and Renilla luciferase activity was measured using the Dual Luciferase Reporter Assay System (Promega; Madison, WI, USA)48 h post-transfection. All measurements were repeated in triplicate.

### Animal experiments

To evaluate the pulmonary metastatic ability of NPC cells in vivo, 100 μl of SUNE-1-BART8-3p cells at a density of 1.0 × 10^6^ cells/ml or an equal amount of control cells were intravenously injected into the tail vein of the nude mice. All mice were subjected to fluorescent imaging on anLT-9MACIMSYSPLUS whole-body imaging system (Lighttools Research; Encinitas, CA, USA) 6-weeks post-injection and then sacrificed. Pulmonary metastatic lesions were sampled and quantified.

### Statistical analysis

All statistical analyses were performed using the software SPSS version 24.0 (SPSS, Inc.; Chicago, IL, USA) and Graph Pad Prism 7 (GraphPad; La Jolla, CA, USA) unless stated specifically. Differences of means were tested for significant significance with Student’s *t*-test, and the correlation between EBV-miR-BART8-3p expression and RNF38 mRNA expression was evaluated with Spearman’s correlation analysis. A *P* value less than 0.05 was considered statistically significant.

## Results

### EBV BART miRNAs were highly up-regulated and co-expressed in NPC

**Fig. 1:**
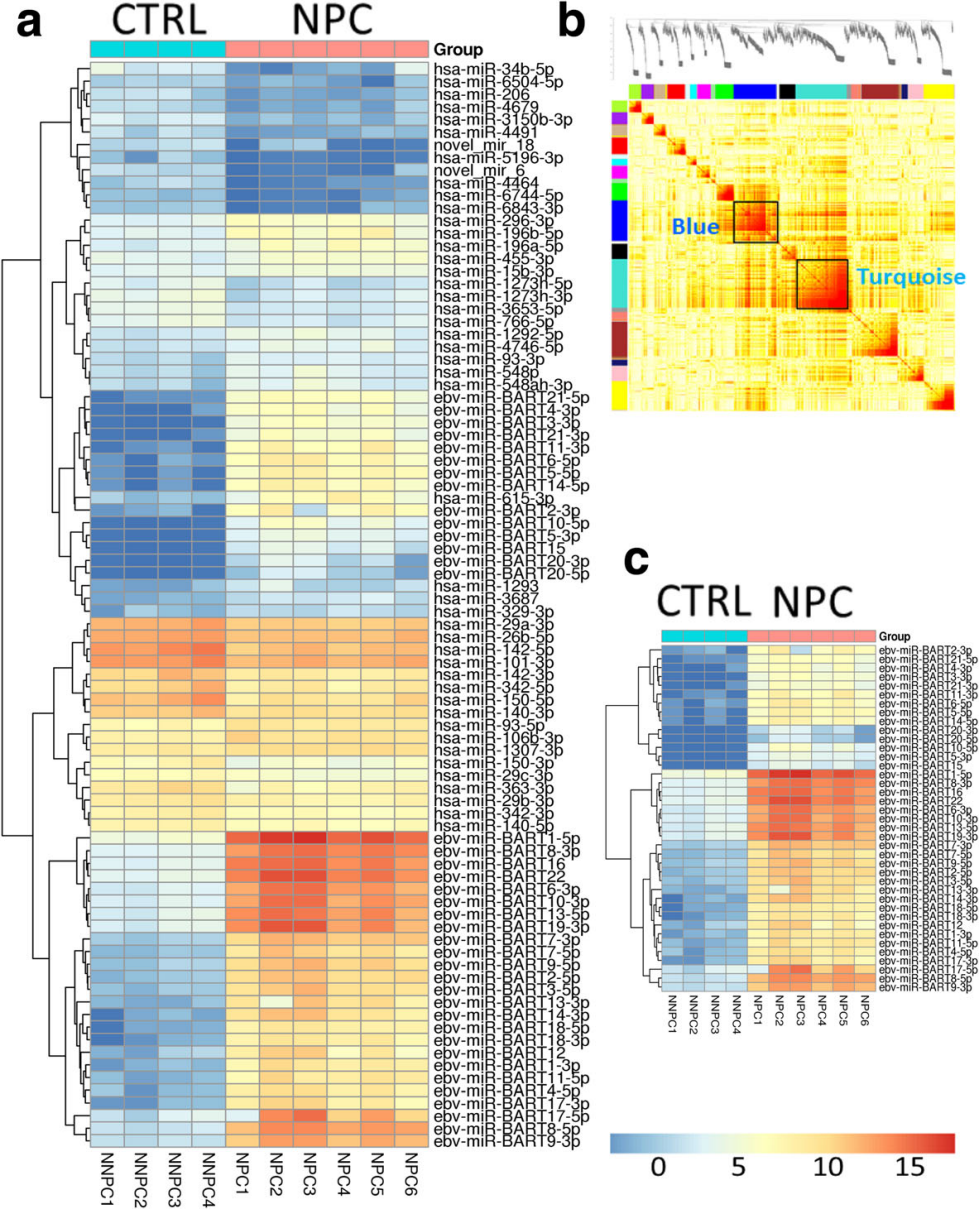
EBV-miR-BART8-3p expression is upregulated and co-expressed in NPC. **a** Heat map of 86 miRNAs differentially expressed between NPC specimens and normal nasopharyngeal mucosal specimens (CTRL). **b** Topological overlap matrix (TOM) plot of the microRNA co-expression network. The x- and y-axes represent 1315 microRNAs and the color intensity indicates interaction strength between microRNAs with red for the strongest interaction and white for no interaction. The color bars along the x- and y-axes denote the module membership. **c** The 20 most highly upregulated EBV BART miRNAs identified between NPC specimens and normal nasopharyngeal mucosal specimens

We first performed differential expression analysis of the miRNA sequencing data from 6 human NPC biopsy specimens and 4 normal nasopharyngeal mucosal specimens (termed control) to systematically uncover miRNAs associated with NPC pathogenesis. The clinical data of NPC cases are showed in Table 1 and TNM stage was listed according to The Union for International Cancer Control / The American Joint Committee on Cancer (UICC/AJCC) 7th of NPC. This dataset has 1315 miRNAs which includes 40 EBV BART miRNAs and 2 EBV BHRF1 miRNAs (i.e., EBV-miR-BHRF1–1 and EBV-miR-BHRF1–2-3p). We identified 30 downregulated miRNAs and 56 upregulated miRNAs in NPC versus control using the criteria of an FC of > 1.2 and an FDR of < 0.05 (Fig. 1a; Additional file 1: Table S1). The two EBV BHRF1 miRNAs and one EBV BART miRNA (EBV-miR-BART19-5p) were not differentially expressed but all the rest 39 differentially expressed EBV BART miRNAs were all upregulated and were highly significantly enriched in the overall 56 upregulated miRNAs (corrected Fisher’s Exact Test (FET) *p* = 9.63E-60, 22.90 fold enrichment (FE)). Interestingly, the top 35 most significantly upregulated miRNAs identified were all EBV BART miRNAs (Additional file 1: Table S2), indicating that EBV BART miRNAs may play an important role in the development of NPC.

**Table 1:** The clinical data of six NPC samples for miRNA sequencing

Number	Sex	Age	Histology	T	N	M	TNM	EBV
NPC1	Male	63	Nonkeratinizing undifferentiated	4	3	0	IVb	**+**
NPC2	Female	21	Nonkeratinizing undifferentiated	3	3	0	IVb	**+**
NPC3	Male	23	Nonkeratinizing undifferentiated	2	1	0	II	**+**
NPC4	Male	61	Nonkeratinizing undifferentiated	1	3	1	IVc	**+**
NPC5	Female	24	Nonkeratinizing undifferentiated	3	1	0	III	**+**
NPC6	Female	46	Nonkeratinizing undifferentiated	2	1	0	II	**+**

Abbreviations: *NPC* nasopharyngeal carcinoma, *EBV* Epstein-Barr virus

To explore the relationship among the miRNAs, especially the EBV BART miRNAs, we applied the well-established weighted gene co-expression network analysis (WGCNA) [33, 36–40] to the whole miRNA sequencing data. Fig. 1b shows a heat map of the weighted miRNA co-expression network. Twenty five modules with sizes between 3 and 206 microRNAs were identified (Additional file 1: Table S3). Only the turquoise and blue modules are enriched for differentially expressed miRNAs (DEMs) in NPC versus control. Note that the turquoise module comprised of 206 microRNAs includes 39 of all the 40 EBV BART microRNAs (corrected FET *p* = 1.32E-30, 6.2 FE). The only EBV BART miRNA that is not in the turquoise module is EBV-miR-BART19-5p, the only EBV BART miRNA was not differentially expressed in NPC versus control. Interestingly, the blue module is only enriched for the downregulated miRNAs (corrected FET *p* = 0.014, 3 FE) while the turquoise module is enriched for both upregulated (corrected FET *p* = 2.18E-44, 6.27 FE) and down-regulated ones (corrected FET *p* = 1.52E-5, 3.6 FE), as shown in Additional file 1: Table S4.

### EBV BART miRNAs are associated with deactivated immune response in NPC

To understand the downstream pathways regulated by miRNAs that may potentially drive NPC pathogenesis, we sought out to analyze the matched transcriptomic data from the same set of samples to identify DEGs in NPC versus control. We identified 2294 downregulated and 1286 upregulated genes in NPC versus control (Additional file 1: Table S5) at FDR ≤ 0.05 and FC ≥ 1.2. The genes upregulated in NPC were most enriched for the cell cycle related functional ontology terms (2.9-FE, corrected FET *P* = 2.21E-31),while those downregulated in NPC versus normal control were most enriched for immune response related pathways including lymphocyte activation (3.3-FE, corrected FET *P* = 1.91E-20), immune system process (1.8-FE, corrected FET P 1.75E-18), leukocyte activation (3-FE, corrected FET *P* = 1.75E-18), cell activation (2.6-FE, corrected FET *P* = 6.59E-18), and immune response (2.0-FE, corrected FET *P* = 4.59E-18), as shown in Additional file 1: Table S6 and Additional file 2: Figure S1.

Meanwhile, we identified the genes significantly negatively correlated with each miRNA. These miRNA-correlation gene (MCG) signatures were then tested for enrichment of the DEG signature between NPC and normal control. Surprisingly, the MCG signatures for all the EBV BART microRNAs up-regulated in NPC versus control were highly significantly enriched for the DEGs (corrected FET *P* < 1.53E-272, FE > 6.1; Additional file 1: Table S7), suggesting that the activation of EBV BART miRNAs dramatically shut down the immune defense system in NPC.

### EBV-miR-BART8-3p is upregulated in human NPC specimens

**Fig. 2:**
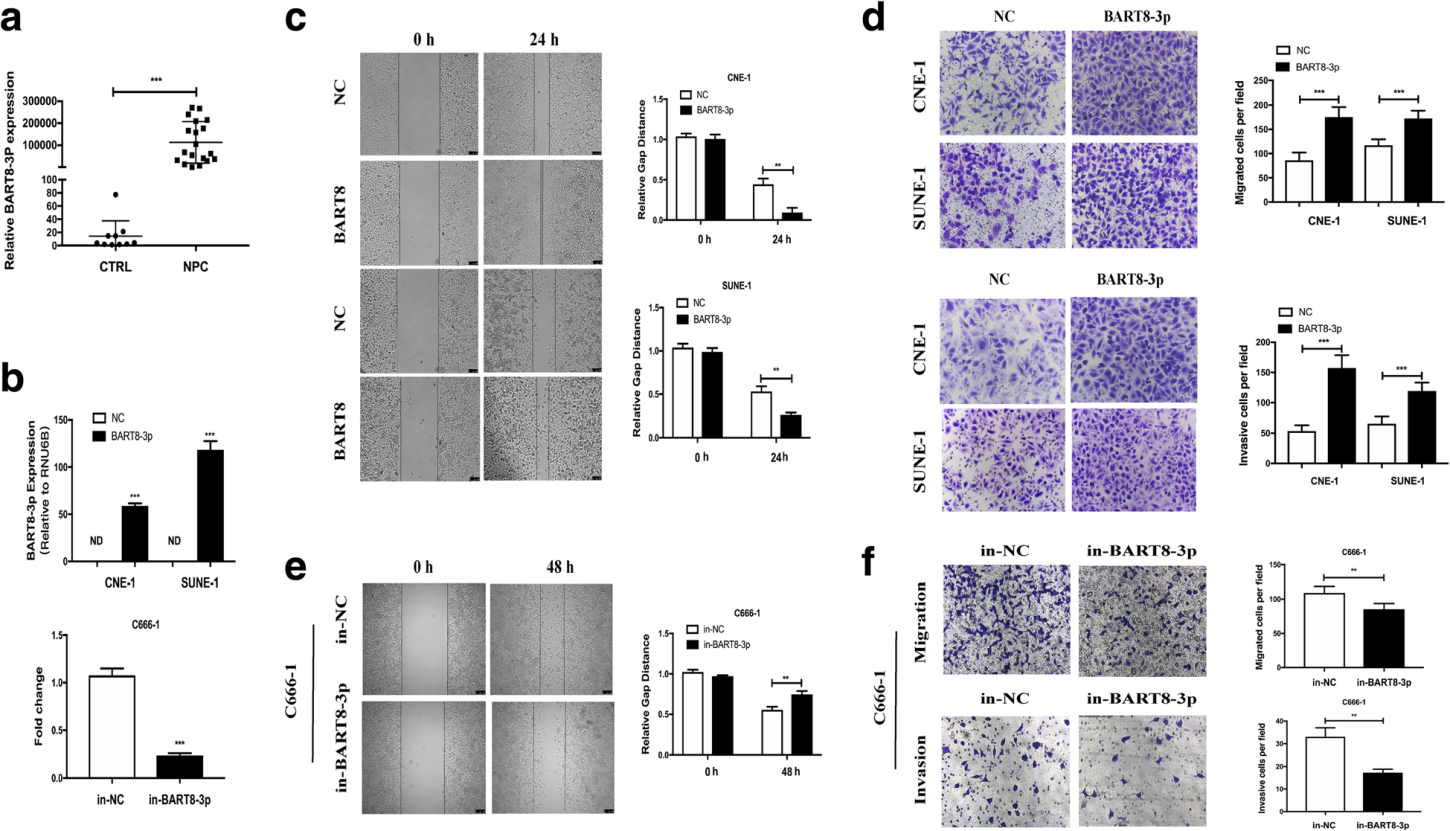
EBV-miR-BART8-3p promotes NPC cell migration and invasion in vitro and lung metastasis in vivo. **a** qPCR assay reveals higher EBV-miR-BART8-3pexpression in NPC specimens than in normal nasopharyngeal mucosal specimens (CTRL). **b** qPCR assay reveals higher EBV-miR-BART8-3p expression in NPC cells transfected withEBV-miR-BART8-3p precursors than in those transfected with control vectors. miRNA levels are normalized to U6 snRNA expression. **c** Representative images and quantification of the wound-healing assay in CNE-1 andSUNE-1cells. **d** Representative images and quantification of migration and invasion assays in CNE-1 andSUNE-1. **e** Representative images and quantification of the wound-healing assay in C666–1 cells. **f** Representative images and quantification of migration and invasion assays in C666–1 cells. ND, not detectable. Data is presented as the mean ± SD. ** *P* < 0.01; *** *P* < 0.001

EBV-miR-BART8-3p is the most up-regulated EBV BART miRNAs in NPC **(**FC = 1782.9, *p* = 4.24E-12, FDR = 5.48E-09; Fig. 1c**,** Additional file 1: Table S2). To confirm BART8-3p expression by RNA sequencing, we further examined BART8-3p expression in NPC and normal nasopharyngeal mucosal specimens (CTRL). Data indicated that BART8-3p was nearly undetectable in CTRL, while strongly over-expressed in NPC (Fig. 2a). Of note, among top 10 upregulated EBV BART miRNAs, BART 16, 7, 9, 2, 1, 22, 3 and 6, have been reported to be associated with the development of NPC [41]. BART8–5 was suggested to cause progression of nasal NK-cell lymphoma [42]. We wonder whether BART8-3p plays a role in the carcinogenesis of NPC. Based on miRNA sequencing results and previous studies, we focused on exploring potential function of BART8-3p in NPC.

### EBV-miR-BART8-3p promotes NPC migration, invasion and metastasis in vitro and in vivo

To investigate the function of EBV-miR-BART8-3p in NPC, two EBV-negative NPC cell lines CNE-1 and SUNE-1 were transfected with lentiviral vectors containing EBV-miR-BART8-3p precursors, and qPCR assay showed higher EBV-miR-BART8-3p expression in NPC cells transfected with EBV-miR-BART8-3p precursors than in those transfected with control vectors (*P* < 0.001) (Fig. 2b). Wound-healing and migration and invasion assays revealed that upregulation of EBV-miR-BART8-3p expression promoted NPC cell migration (*P* < 0.01) and invasion (*P* < 0.001) compared to control vectors-transfected cells (Fig. 2c and d), while downregulation of EBV-miR-BART8-3p suppressed EBV-positive NPCC666–1 cell migration (*P* < 0.01) and invasion (*P* < 0.01) (Fig. 2e and f). However, no significant differences were detected between GV369- or GV280-transfected NPC cells and control vectors-transfected cells in terms of cell proliferation or cell colonies (Additional file 2: Figure S2).

**Fig. 3:**
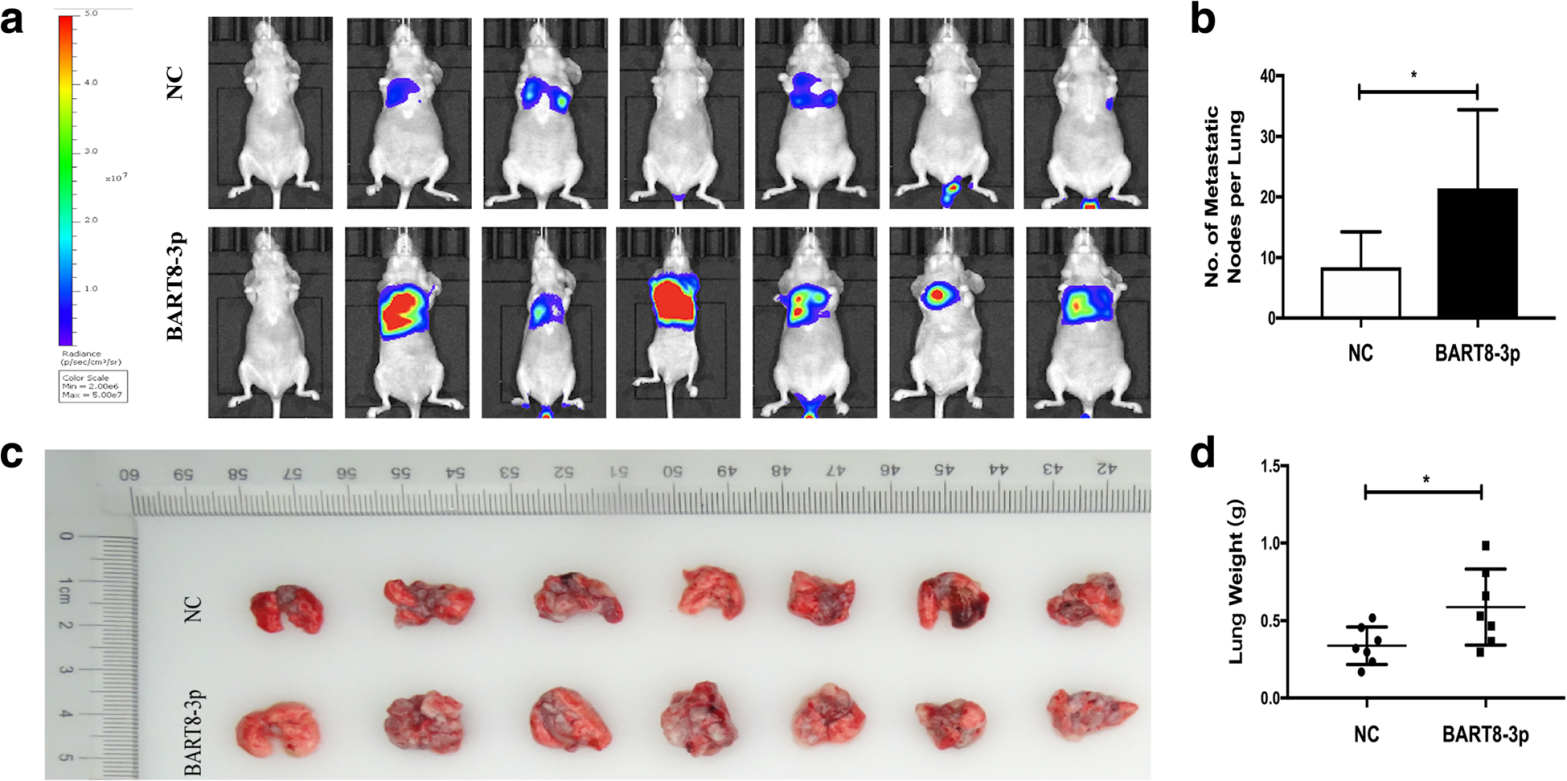
Overexpression of EBV-miR-BART8-3p promotes lung metastasis of NPC in vivo. **a** Nude mice were intravenously injected with SUNE-1-BART8-3p cells or control vector-transfected SUNE-1 cells via the tail veins, and were sacrificed 6 weeks post-injection. Representative images in vivo were obtained by the whole-body imaging system. **b** Representative images of metastatic nodules in mouse lungs. **c** Number of metastatic nodules in mouse lungs. **d** Weight of mouse lungs. *P < 0.05

To assess the pulmonary metastatic ability of EBV-miR-BART8-3p in vivo, we modeled pulmonary metastasis of NPC in nude mice by inoculation of SUNE-1-BART8-3p cells or control vector-transfected SUNE-1 cells into the tail veins of nude mice. We found more metastatic tumor nodules in the lung of mice injected with SUNE-1-BART8-3p cells than injection with control vector-transfected SUNE-1 cells (*P* < 0.05) (Fig. 3a and b), and a greater weight of lungs was measured in mice inoculated with SUNE-1-BART8-3p cells than injection with control vector-transfected SUNE-1 cells (*P* < 0.05) (Fig. 3c and d). Taken together, these results suggest that EBV-miR-BART8-3p upregulation promotes NPC cell migration, invasion and metastasis.

### EBV-miR-BART8-3p upregulation promotes epithelial-mesenchymal transition (EMT) and increases metastasis-related markers expression

**Fig. 4:**
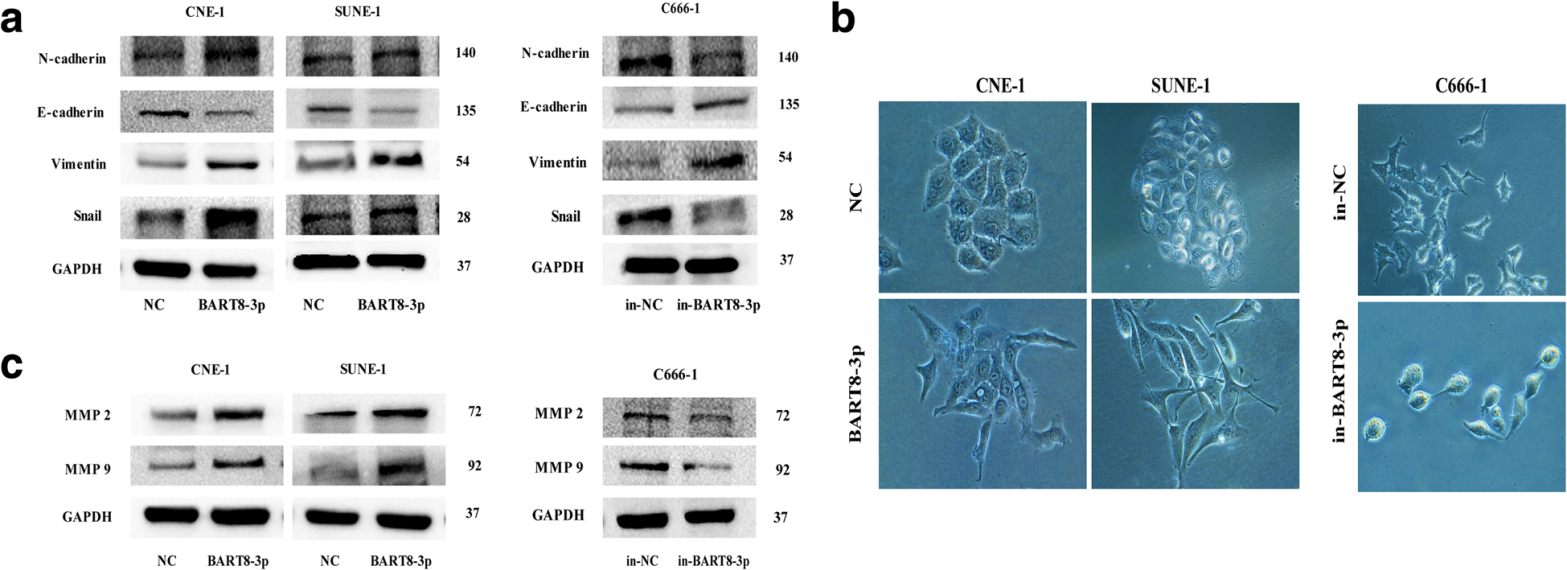
EBV-miR-BART8-3pregulatesEMT and increases metastasis-related markers expression in NPC cells. **a** Western blotting reveals that upregulation of EBV-miR-BART8-3p results in a reduction in the E-cadherin expression and an increase in the Snail, N-cadherin, Vimentin expression. GAPDH serves as an internal control. **b** Morphology changes are observed using phase contrast microscopy (magnification, × 200). **c** Western blotting reveals that upregulation of EBV-miR-BART8-3presults in an increase in the MMP2 and MMP9 expression

Since EMT is closely associated with NPC metastasis [14, 15], we then examined the role of EBV-miR-BART8-3p in EMT of NPC cells. The expression of EMT- and metastasis-related markers was determined in CNE-1 and SUNE-1 cells. Western blotting showed that upregulation of EBV-miR-BART8-3p caused a reduction in the E-cadherin expression and an increase in the Snail, N-cadherin, Vimentin, MMP2 and MMP9 expression (Fig. 4b). In addition, the NPC cells overexpressing EBV-miR-BART8-3p displayed a mesenchymal-like morphology, while control vectors-transfected NPC cells showed an epithelial-like morphology (Fig. 4b).

Conversely, downregulation of EBV-miR-BART8-3p had the opposite effects in EBV-positive NPC C666–1 cells. EBV-miR-BART8-3p knockdown resulted in a reduction in Snail, N-cadherin and Vimentin expression and an increase in E-cadherin expression, and impeded the EMT-like process in NPC cells (Fig. 4). Taken together, our findings suggest that EBV-miR-BART8-3p drives an EMT process and upregulates metastasis-related proteins in NPC cells.

### EBV-miR-BART8-3p induces NPC cell metastasis by activating the NF-κB and Erk1/2 pathways

**Fig. 5:**
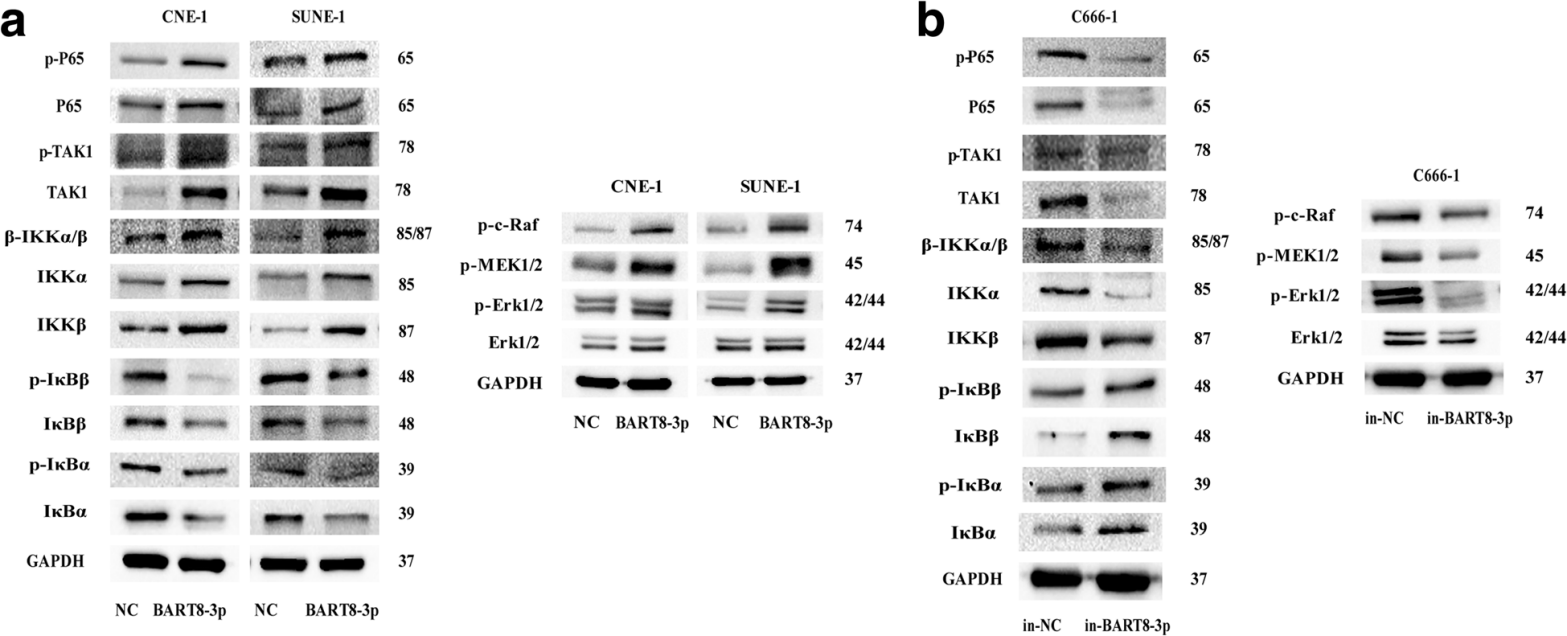
EBV-miR-BART8-3p regulates NF-κB and Erk1/2 signaling pathways. **a** Upregulation of EBV-miR-BART8-3p activates NF-κB and Erk1/2 signaling pathways in CNE-1 and SUNE-1 cells. **b** Downregulation of EBV-miR-BART8-3p inhibits NF-κB and Erk1/2 signaling pathways in C666–1 cells

To address the mechanisms underlying miR-BART8-3p-driving metastasis in NPC, we determined the expression of key proteins in the NF-κB and Erk1/2 signaling pathways. Western blotting showed that upregulation of EBV-miR-BART8-3p increased the total and phosphorylation expression of TAK1, p65, IKKα, IKKβ and IKKα/β, and reduced the IκBβ, p-IκBβ, IκBα and p-IκBα expression; in addition, the Erk1/2, p-c-Raf, p-MEK1/2 and p-Erk1/2 expression was also elevated following EBV-miR-BART8-3p upregulation (Fig. 5a). On the contrary, downregulation of EBV-miR-BART8-3p resulted in opposite effects in C666–1 cells (Fig. 5b). Collectively, our data indicate that EBV-miR-BART8-3p mediates NPC cell metastasis through activating the NF-κB and Erk1/2 signaling pathways.

### EBV-miR-BART8-3p directly targets *RNF38* in NPC cells

**Fig. 6:**
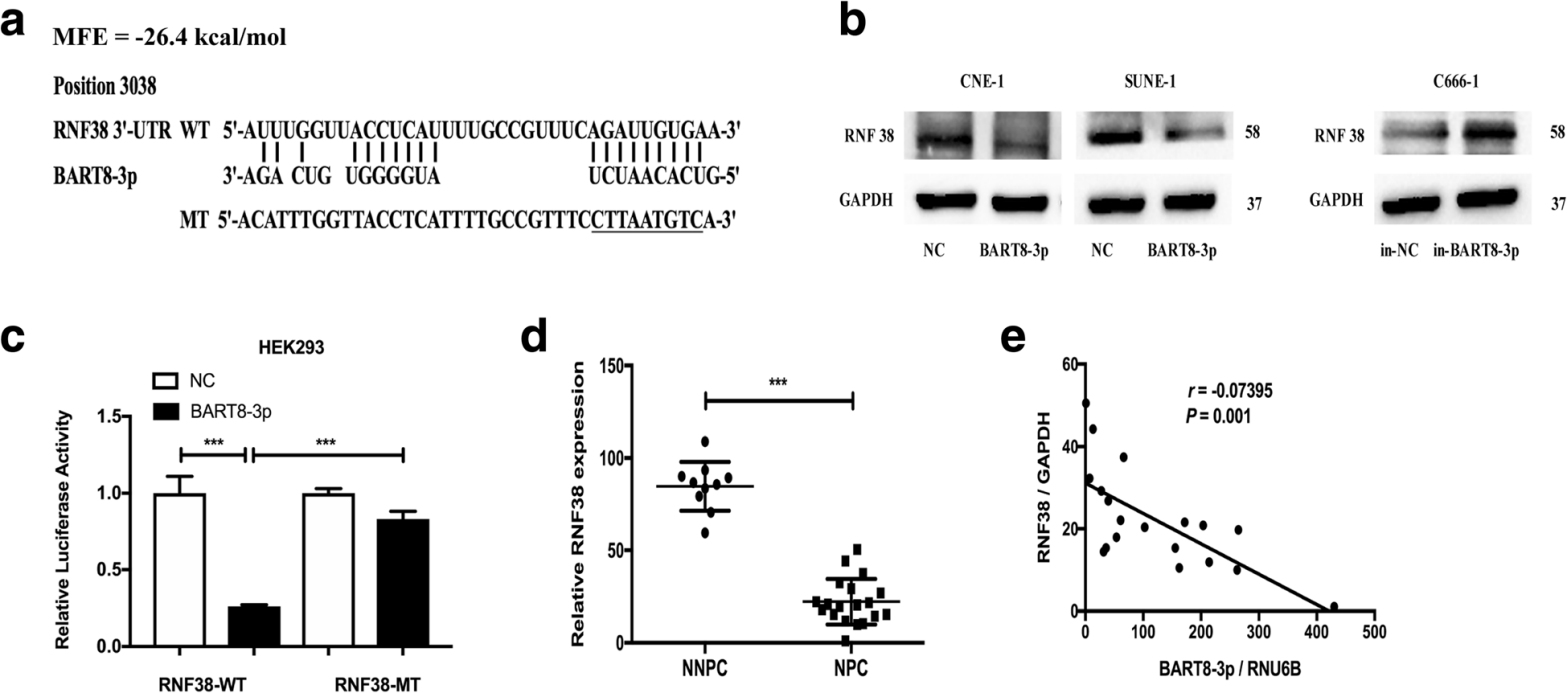
RNF38 is a direct target of EBV-miR-BART8-3p. **a** EBV-miR-BART8-3p and its putative binding sequence in 3’UTR of RNF38 mRNA, and mutations are generated as indicated. **b** Quantification of RNF38protein expression by Western blotting in CNE-1, SUNE-1 and C666–1 cells. **c** The relative luciferase activity in HEK293 cells after co-transfection with wild-type (WT) or mutant (MT) RNF38 3’UTR reporter genes and EBV-miR-BART8-3p or control. **d** Relative RNF38 expression is quantified in 10 normal nasopharyngeal specimens and 19 NPC specimens by qPCR, and GAPDH serves as an internal control. **e** Spearman’s correlation analysis reveals a negative correlation between the EBV-miR-BART8-3p and RNF38 expression in NPC specimens (*n* = 19)

Then, we predicted the target genes of EBV-miR-BART8-3p. Bioinformatics analysis showed that the 3′-UTR region of the gene *RNF38* was complementary with the seed sequences of EBV-miR-BART8-3p and *RNF38* had the lowest MFE (Fig. 6a). Firstly, we quantified endogenous RNF38 protein expression using Western blotting, and the RNF38 protein was found to be significantly downregulated in both CNE-1-BART8-3p and SUNE-1-BART8-3p cells (Fig. 6b). Subsequently, we cloned the wild-type or mutant-type 3′-UTR of *RNF38* into luciferase reporter vectors, and the luciferase activity was found to be reduced significantly in HEK293 cells transfected with the wild-type 3′-UTR of RNF38 (*P* < 0.001), while no changes were seen in the cells transfected with the mutant-type (Fig. 6c), suggesting that RNF38 is regulated by EBV-miR-BART8-3p.

To further quantify the RNF38 expression, we determined *RNF38* mRNA expression in 19 NPC specimens and 10 normal nasopharyngeal specimens, and examined the correlation between EBV-miR-BART8-3p and *RNF38* expression in the19 NPC specimens. qPCR assay showed significantly lower RNF38 expression in NPC specimens than in normal nasopharyngeal specimens (*P* < 0.001) (Fig. 6d), and Spearman’s correlation analysis revealed a negative correlation between EBV-miR-BART8-3p and *RNF38* expression (*P* = 0.001) (Fig. 6e). Taken together, these data indicate that EBV-miR-BART8-3p inhibits *RNF38* expression in NPC through directly targeting *RNF38*.

### Restored *RNF38* rescues the phenotypes induced by EBV-miR-BART8-3p overexpression

**Fig. 7:**
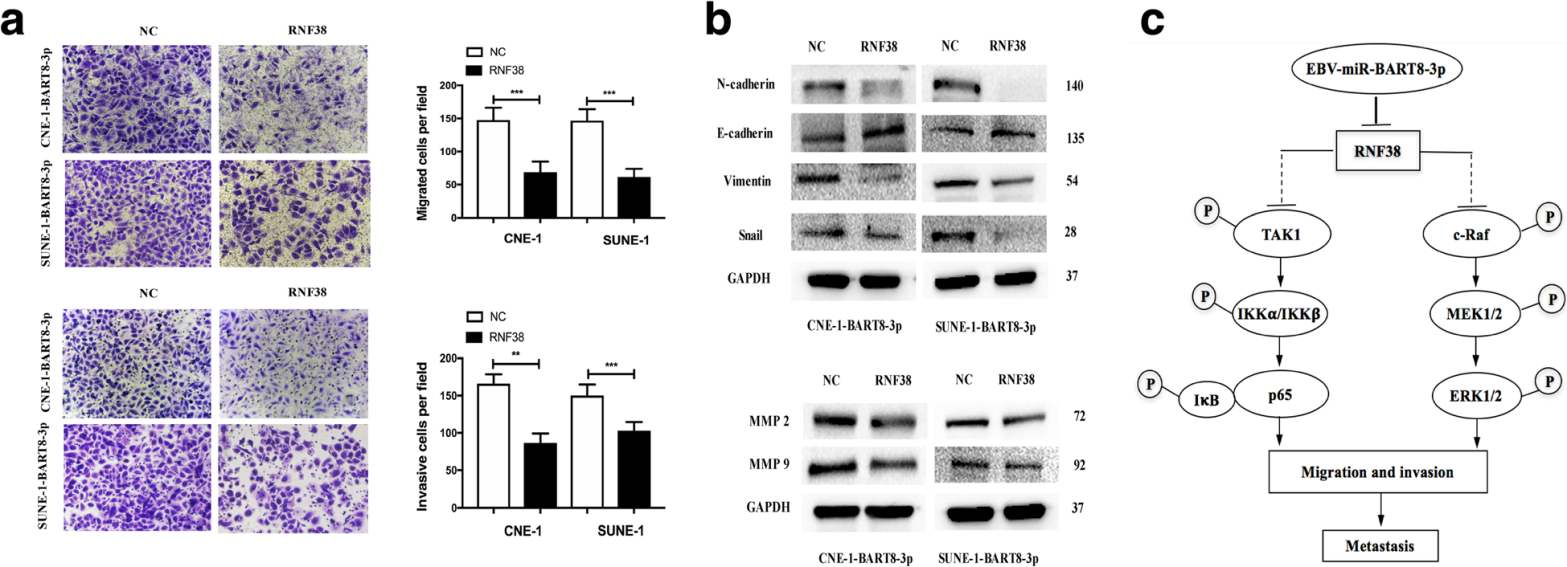
Restored RNF38 rescues the phenotypes of NPC cells. **a** Reconstitution of RNF38 reduces the migration and invasion of CNE-1-BART8-3p and SUNE-1-BART8-3p cells. **b** Reconstitution of RNF38 reverses the expression of EMT-associated markers and metastasis-related proteins. **c** The proposed model shows the role of EBV-miR-BART8-3p in regulation of NPC metastasis. **P < 0.01; *** *P* < 0.001

To further confirm the functions of *RNF38* in the tumor-promoting effects of EBV-miR-BART8-3p, we rescued RNF38 expression in CNE-1-BART8-3p and SUNE-1-BART8-3p cells. The introduction of exogenous *RNF38* significantly reduced NPC cell migration and invasion (Fig. 7a). Consistent with this biological phenotype, reinstallation of RNF38 reversed EMT and expression of metastasis-related markers by EBV-miR-BART8-3p (Fig. 7b). These data strongly support our hypothesis that *RNF38* is an important mediator of EBV-miR-BART8-3p-induced NPC cell migration and invasion (Fig. 7c).

## Discussion

EBV infection has been proved to be strongly associated with a variety of lymphoid and epithelial malignancies [3], notably NPC. Since the EBV-miRNAs were firstly discovered in 2004, there are 44 mature EBV BART miRNAs identified to date [43]. Our report showed that a vast majority (97.5% or 39) of the 40 EBV BART miRNAs profiled in this study were significantly upregulated in NPC and further study suggested that these upregulated EBV BART miRNAs were all co-expressed in the same gene module according to WGCNA. In addition, the analysis of DEGs and MCG signatures revealed that EBV BART miRNAs may be involved in immune response, which is little known until now [19, 20].

So far, a few EBV BART miRNAs have been investigated and partially defined in NPC. EBV-miR-BART20-5p, EBV-miR-BART5 and EBV-miR-BART cluster I (miR-BART1-5p, miR-BART16 and miR-BART17-5p) were reported to inhibit apoptosis of NPC cells by targeting BAD, PUMA and LMP1, respectively [9, 10, 13] while miR-BART22 and miR-BART2-5p have been found to contribute to immune evasion through targeting LMP2A and MICB, respectively [19, 20]. Additionally, miR-BART18–5p and miR-BART2 have shown regulatory effects on viral latency by inhibiting MAP3K2 and BALF5 [22, 23]. However, there is little knowledge on the roles of EBV BART miRNAs in metastasis of NPC [26], and the oncogenic and tumor suppressive activities of EBV BART miRNAs remain unknown. Previous studies have demonstrated that miR-BART1, miR-BART7-3p, miR-BART9 and miR-BART10-3p induce tumor metastasis by targeting PTEN, E-cadherin and BTRC, respectively [14–17]. However, the role of EBV-miR-BART8-3p is still unknown in NPC. Our findings showed that EBV-miR-BART8-3p was the most upregulated EBV BART miRNA and it contributed to NPC metastasis by targeting *RNF38*. A recent study reported that miR-BART6-3p inhibits NPC cell metastasis and invasion by targeting long non-coding RNA [18]. Therefore, like human miRNAs, EBV BART miRNAs also constitute a complex miRNA regulatory network and have shown both positive and negative effects on metastatic progression in NPC [44]. It is therefore of great significance to investigate how EBV BART miRNAs are generated and whether they act as oncogenes or tumor suppressors in metastatic progression.

EMT is considered to be a key part of tumor migration and invasion [45]. Until now, few studies have shown the crucial role of EBV BART miRNAs during the process of EMT in NPC, including EBV-miR-BART1, EBV-miR-BART7-3p, EBV-miR-BART9 and EBV-miR-BART10-3p [14–17]. To our knowledge, the present study is the first report demonstrating that EBV-miR-BART8-3p upregulation facilitated EMT, leading to the metastasis of NPC cells. In addition, upregulation of EBV-miR-BART8-3p increased the expression of EMT-related markers and metastasis-related proteins, while downregulation of EBV-miR-BART8-3p attenuated EMT and reduced the expression of EMT-associated proteins. Further studies, however, to investigate the proportions of EBV BART miRNAs and the mechanisms underlying the role of EBV BART miRNAs during the process of EMT in NPC seem justified.

Increasing evidence suggests that NF-κB signaling plays a crucial role in maintaining EBV latency in NPC cells through regulating EBV BART miRNAs and lncRNAs [46], and NF-κB activation is essential in the pathogenesis and progression of NPC [47, 48]. Moreover, NF-κB and Erk1/2 signaling pathways have been found to be vital for EMT and metastasis, along with the upregulation of MMPs [45, 49–51]. It is therefore hypothesized that NF-κB and Erk1/2 signaling is involved in the process of EBV-miR-BART8-3p-induced metastasis in NPC. Our data supported our hypothesis that upregulation of EBV-miR-BART8-3p induced NPC metastasis by activating the NF-κB and Erk1/2 signaling pathways, while downregulation of EBV-miR-BART8-3p had the opposite effects. Of note, previous studies have shown that EBV-miR-BART1 activates PI3K-AKT, FAK-p130^Cas^ and MAPK-ERK1/2 pathways, miR-BART10-3p activates β-catenin/Snail signaling and miR-BART7 activates PI3K/AKT and p-GSK-3β-ser9 signaling to promote NPC metastasis [14–16]. In agreement with EBV-miR-BART1, we found that Erk1/2 signaling was closely associated with the metastasis in NPC. Interestingly, we also found, for the first time, that NF-κB signaling contributed to EBV-miR-BART8-3p-induced NPC metastasis. Further studies to examine the mechanisms underlying the contribution of NF-κB signaling to the metastasis of NPC are warranted.

RING finger protein family is involved in a variety of diverse biological processes, including oncogenesis, signal transduction, apoptosis, development and viral infection [52]. *RNF38*, located at chromosome 9 (9p 13), is a member of the RING finger protein family [52]. It is reported that chromosome 9p loss was a key event during the pathogenesis of NPC and frequently deleted in multiple cancers, such as lung cancer [53, 54], hepatocellular carcinoma [55], squamous cell head and neck cancer [56] and NPC [57]. Previous studies have shown that RNF38 might modify p53 [58, 59] and was associated with the neuronal activity [60]. However, the role of *RNF38* in cancer remains unknown until now. Our data showed that RNF38 might act as a tumor suppressor in NPC.

Our previous study reported that circulating EBV BART miRNAs might be used as biomarkers for early diagnosis and prognosis of NPC [25]. Therefore, we hypothesize that EBV-miR-BART8-3p may be predictive of prognosis in NPC. Further understanding of the role and the potentially clinical value of *RNF38* in the carcinogenesis of NPC and the potential clinical value of EBV-miR-BART8-3p in NPC requires further investigations. Since a growing number of miRNAs and their targeting genes have been identified [61] and they form complex regulatory miRNA network as we demonstrated here, further study of regulatory relationships between EBV-miR-BART8-3p and its target genes in NPC are warranted. Moreover, future studies should also examine the relationships between upregulated EBV BART miRNAs and deactivated immune response genes as well as the relationships between up-regulated genes and cell cycle related processes in NPC.

## Conclusions

The results of the present study demonstrate that EBV-miR-BART8-3p is upregulated in human NPC specimens, and EBV-miR-BART8-3p overexpression facilitates EMT, invasion and migration through directly targeting *RNF38* in NPC cells, via the activation of NF-κB and Erk1/2 signaling pathways. Our findings suggest that EBV-miR-BART8-3p may be a promising therapeutic target for NPC.

## Additional files

Additional file 1: **Table S1.** miRNAs differentialy expressed in NPC versus control. **Table S2.** The top 20 most significantly up-regulated microRNAs in NPC. **Table S3.** Module assignment for microRNAs. **Table S4.** Enrichement of differentially expressed microRNAs in the coexpressed microRNA module. **Table S5.** Differerentially expressed genes in NPC versus control (NNPC). **Table S6.** Gene ontology and pathways enriched in the up- and down-regulated genes. The functional annotation gene sets were from the MSigDB database. FE, fold enrichment. Pvalue, *P* value was computed from Fisher’s exact test. P.adj, adjusted P value computed with Benjamini-Hochberg’s false discovery rate approach. **Table S7.** Enrichement of the differentially expressed gene signature in the microRNA-correlated gene signatures. (XLSX 732 kb)

Additional file 2: **Figure S1.** Gene ontology terms enriched in the up- and down-regulated gene signatures. The genes up-regulated in NPC are involved in cell cycle, neurogenesis and cell junction activities while those down-regulated are associated with immune response, suggesting activated cell proliferation and mitosis but inhibited immune defense in NPC. **Figure S2.** Upregulation of EBV-miR-BART8-3p shows no clear-cut effects on NPC cell proliferation in vitro. a, the effect of EBV-miR-BART8-3p on NPC CNE-1 and SUNE-1 cell proliferation is examined by CCK-8 assay; b, representative pictures (left panel) and quantification (left panel) of the colony-forming assays in CNE-1 and SUNE-1 cells. NS, no significant. Data are presented as mean ± SD. (DOCX 957 kb)

## Abbreviations

AJCC: The American Joint Committee on Cancer
BART: BamHI-A rightward transcripts
BHRF1: *Bam*HI fragment H rightward open reading frame 1
DEMs: differentially expressed miRNAs
EBERs: EBV-encoded RNAs
EBNA1: EBV nuclear antigen 1
EBV: Epstein-Barr virus
EBV-miRNAs: EBV-encoded microRNAs
EMT: Epithelial-mesenchymal transition
FC: Fold change
FDR: False discovery rate
FET: Fisher’s Exact Test
GO: Gene ontology
LMP1: Latent membrane proteins 1
LMP2A: Latent membrane proteins 2A
LMP2B: Latent membrane proteins 2B
MCG: miRNA-correlation gene
MFE: Minimum free energy
miRNAs: microRNAs
NPC: Nasopharyngeal carcinoma
TOM: Topological overlap matrix
UICC: The Union for International Cancer Control
WGCNA: Weighted gene co-expression network analysis
