# Correction to: EBV-miR-BART8-3p induces epithelial-mesenchymal transition and promotes metastasis of nasopharyngeal carcinoma cells through activating NF-κB and Erk1/2 pathways

**DOI:** 10.1186/s13046-019-1022-5

**Published:** 2019-01-24

**Authors:** Cheng Lin, Jingfeng Zong, Wansong Lin, Minghui Wang, Yuanji Xu, Rui Zhou, Shaojun Lin, Qiaojuan Guo, Honglin Chen, Yunbin Ye, Bin Zhang, Jianji Pan

**Affiliations:** 1 Fujian Medical University, Fuzhou, 350108 Fujian Province China; 2 Department of Radiation Oncology, Fujian Cancer Hospital & Fujian Medical University Cancer Hospital, Fuzhou, 350011 Fujian Province China; 3 Laboratory of Immuno-Oncology, Fujian Cancer Hospital & Fujian Medical University Cancer Hospital, Fuzhou, 350011 Fujian Province China; 4 Department of Genetics and Genomic Sciences, Icahn Institute of Genomics and Multiscale Biology, Mount Sinai Center for Transformative Disease Modeling, Icahn School of Medicine at Mount Sinai, 1470 Madison Avenue, New York, NY 10029 USA; 5 State Key Laboratory for Emerging Infectious Diseases, Department of Microbiology and the Collaborative Innovation Center for Diagnosis and Treatment of Infectious Diseases, The University of Hong Kong, Hong Kong, SAR China; 6 Fujian Provincial Key Laboratory of Translational Cancer Medicine, Fuzhou, 350014 Fujian China

**Correction to:** ***Journal of Experimental & Clinical Cancer Research*** **(2018) 37:283.**

**Doi 10.1186/s13046-018-0953-6**

Following publication of the original article [1], the authors reported two errors in the article.

- In the caption of Fig. 1c the sentence “The 20 most highly upregulated EBV BART miRNAs identified between NPC specimens and normal nasopharyngeal mucosal specimens” should instead read “The highly upregulated EBV BART miRNAs identified between NPC specimens and normal nasopharyngeal mucosal specimens”.
- In Fig. 3a, the first image in the BART8-3p group was inadvertently imported and replaced with the original one in the NC group. A corrected version of Fig. 3 is shown in this Correction.

**Fig. 3:**
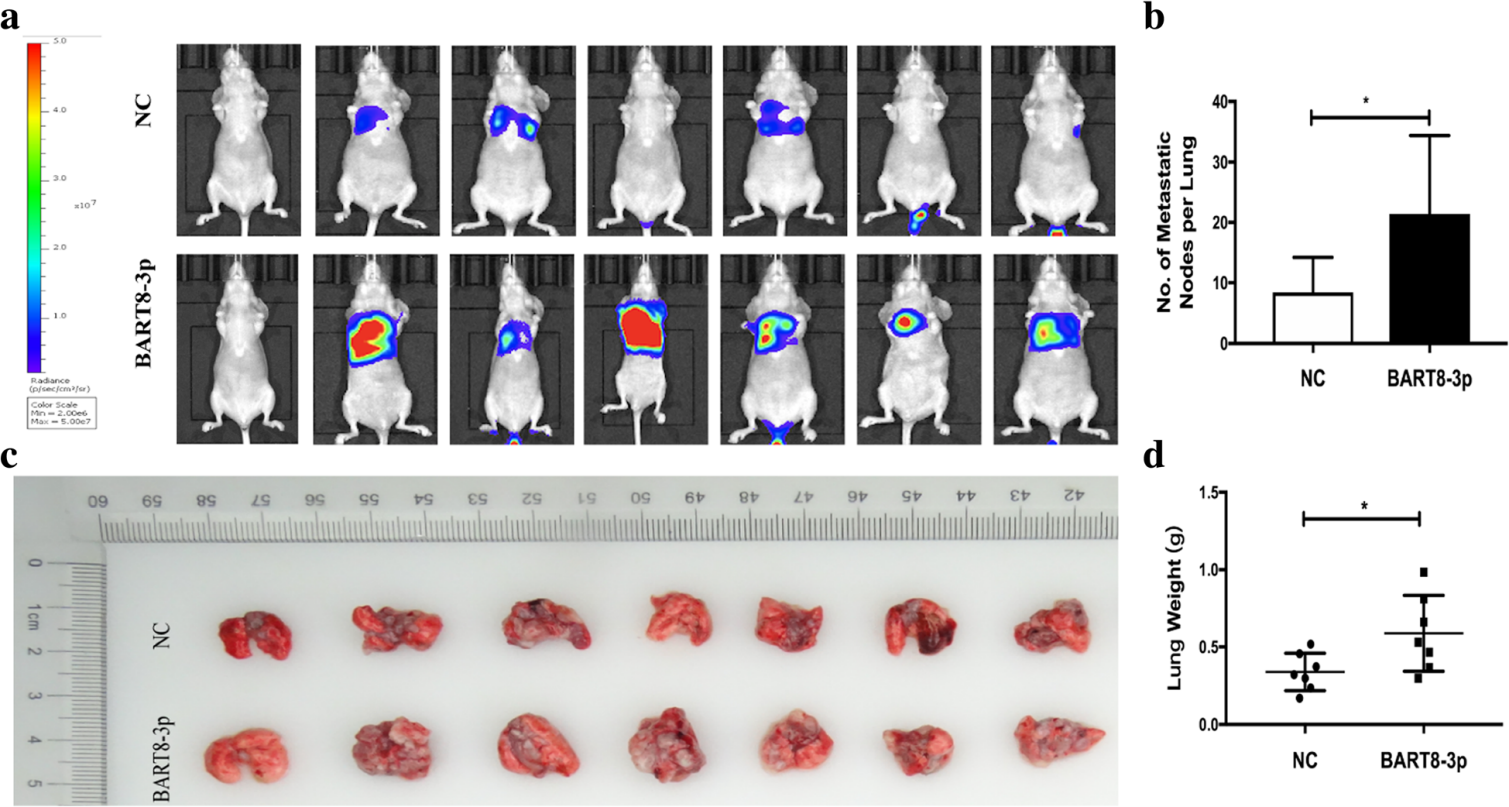
Overexpression of EBV-miR-BART8-3p promotes lung metastasis of NPC in vivo. **a** Nude mice were intravenously injected with SUNE-1-BART8-3p cells or control vector-transfected SUNE-1 cells via the tail veins, and were sacrificed 6 weeks post-injection. Representative images in vivo were obtained by the whole-body imaging system. **b** Representative images of metastatic nodules in mouse lungs. **c** Number of metastatic nodules in mouse lungs. **d** Weight of mouse lungs. *P < 0.05
