# Supplementary material for: EBV-miR-BART8-3p induces epithelial-mesenchymal transition and promotes metastasis of nasopharyngeal carcinoma cells through activating NF-κB and Erk1/2 pathways
Source: J Exp Clin Cancer Res. 2018 Nov 26;37:283. doi: 10.1186/s13046-018-0953-6 (PMC6257964; doi:10.1186/s13046-018-0953-6)
Supplement: Supplementary file 2 — Figure S1. Gene ontology terms enriched in the up- and down-regulated gene signatures. The genes up-regulated in NPC are involved in cell cycle, neurogenesis and cell junction activities while those down-regulated are associated with immune response, suggesting activated cell proliferation and mitosis but inhibited immune defense in NPC. Figure S2. Upregulation of EBV-miR-BART8-3p shows no clear-cut effects on NPC cell proliferation in vitro. a, the effect of EBV-miR-BART8-3p on NPC CNE-1 and SUNE-1 cell proliferation is examined by CCK-8 assay; b, representative pictures (left panel) and quantification (left panel) of the colony-forming assays in CNE-1 and SUNE-1 cells. NS, no significant. Data are presented as mean ± SD. (DOCX 957 kb) [file 13046_2018_953_MOESM2_ESM.docx]

**Additional file 2**

**Cell cycle and immune response pathways were enriched in the genes differentially expressed in NPC versus normal control**

From RNA-seq, 1,286 up-regulated and 2,294 down-regulated genes (**Additional file 1: Table S5**) were identified at false discovery rate (FDR) ≤ 0.05 and fold change (FC) ≥ 1.2. We searched for gene ontology (GO) and functional pathway terms enriched within the differential expression genes (DEGs) signature by making use of the gene sets annotations from the MSigDB database[30]. **Figure S1** shows the fold enrichment (FE) and adjusted P value of the top GO and pathway terms enriched in the up- and down-regulated gene signatures. The complete list of enriched functional categories at an FDR cutoff of 0.05 is provided in **Additional file 1: Table S6**. As expected, cell cycle related functional terms were the mostly enriched category, for example, GO cell cycle showed a FE of 2.9 with FDR adjusted p value= 2.21E-31. Among the GO/pathways enriched in the genes up-regulated in NPC versus normal control include tissue development (2.5-FE, FDR=2.54E-23), cell junction (2.7-FE, FDR=4.94E-21), and embryo development (2.9-FE, FDR=1.41E-20). Among the genes down-regulated in NPC versus normal control, the top enriched GO/pathways are largely immune response related, including lymphocyte activation (3.3-FE, corrected FET P 1.91E-20), immune system process (1.8-FE, corrected FET P 1.75E-18), leukocyte activation (3-FE, corrected FET P 1.75E-18), cell activation (2.6-FE, corrected FET P 6.59E-18), and immune response (2.0-FE, corrected FET P 4.59E-18).


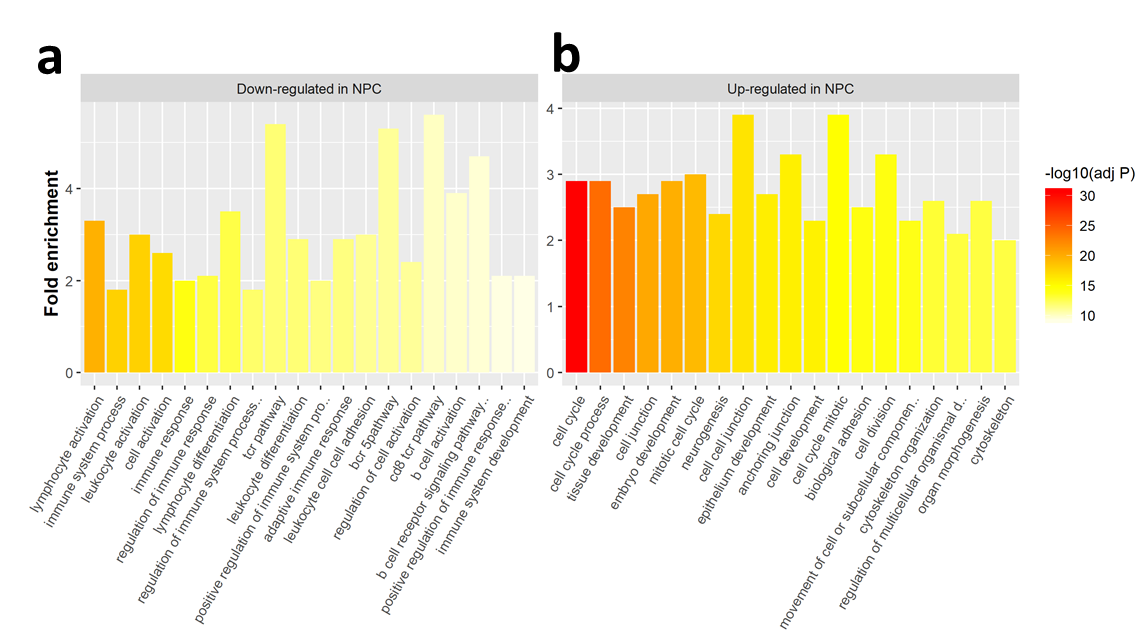


**Figure S1. Gene ontology terms enriched in the up- and down-regulated gene signatures.** The genes up-regulated in NPC are involved in cell cycle, neurogenesis and cell junction activities while those down-regulated are associated with immune response, suggesting activated cell proliferation and mitosis but inhibited immune defense in NPC.


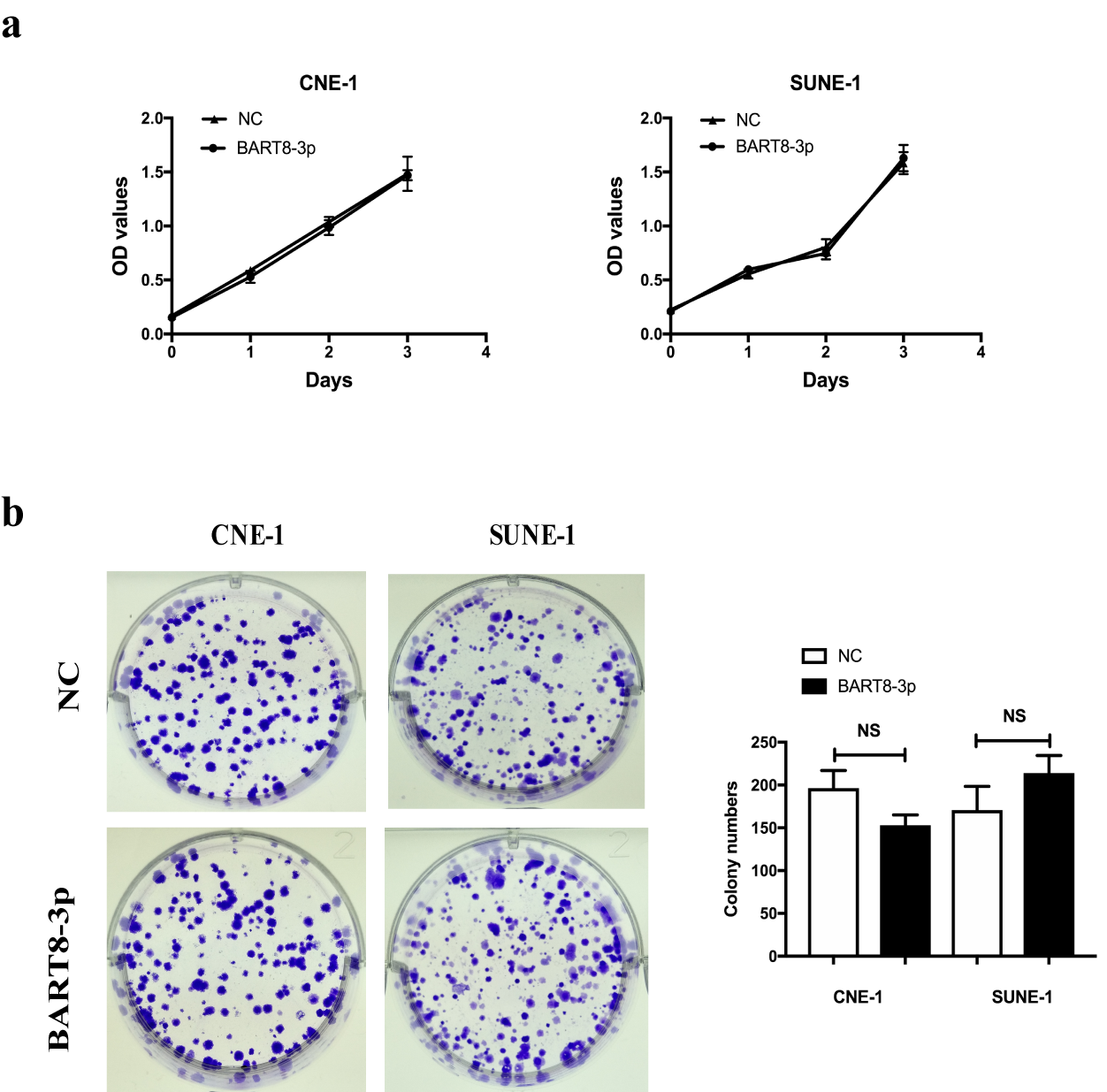


**Figure S2. Upregulation of EBV-miR-BART8-3p shows no clear-cut effects on NPC cell proliferation *in vitro*.** a, the effect of EBV-miR-BART8-3p on NPC CNE-1 and SUNE-1 cell proliferation is examined by CCK-8 assay; b, representative pictures (left panel) and quantification (left panel) of the colony-forming assays in CNE-1 and SUNE-1 cells. NS, no significant. Data are presented as mean ± SD.
